# Efficacy and safety of microneedling with tranexamic acid versus microneedling with vitamin C in the treatment of melasma: a systematic review and meta-analysis

**DOI:** 10.25122/jml-2026-0051

**Published:** 2026-06

**Authors:** Thanapon Roddaje, Sinitta Saowarat, Phawit Norchai

**Affiliations:** 1 College of Integrative Medicine, Dhurakij Pundit University, Bangkok, Thailand

**Keywords:** tranexamic acid, vitamin C, ascorbic acid, microneedling, melasma, hyperpigmentation, systematic review, meta-analysis, CI, confidence interval, EBSCO, Elton B. Stephens Company (database), Hedges' g, standardized mean difference effect size statistic, hemi-MASI, Hemi-Melasma Area and Severity Index, IRB, Institutional Review Board, MASI, Melasma Area and Severity Index, MeSH, Medical Subject Headings, mMASI, Modified Melasma Area and Severity Index, MN, microneedling, PICO, Population, Intervention, Comparator, Outcome (framework), PIH, post-inflammatory hyperpigmentation, PRISMA, Preferred Reporting Items for Systematic Reviews and Meta- Analyses, PROSPERO, International Prospective Register of Systematic Reviews, RCT, randomized controlled trial, RoB 2, Cochrane Risk of Bias Tool Version 2, ROBINS-I, Risk Of Bias In Non-randomized Studies of Interventions, RR, risk ratio, SD, standard deviation, TXA, tranexamic acid, UV, ultraviolet, VEGF, vascular endothelial growth factor

## Abstract

This study aimed to compare the efficacy and short-term tolerability of microneedling combined with either tranexamic acid or vitamin C for melasma. A systematic review and meta-analysis following PRISMA 2020 guidelines was conducted across five databases (MEDLINE, Google Scholar, EBSCO, ProQuest, Web of Science) and ClinicalTrials.gov (2015–2025). Eight studies (232 patients) were synthesized using a random-effects model; outcomes were harmonized across MASI, mMASI, and hemi-MASI using Hedges' g calculated from within-group change-from-baseline scores, with an imputed within-person correlation (r = 0.5; sensitivity range 0.25–0.75) for split-face designs. The pooled analysis showed no statistically significant difference between tranexamic acid and vitamin C (Hedges' g = −0.123; 95% CI, −0.311 to 0.065; *P* = 0.201; I^2^ = 0.0%). Exploratory subgroup analyses by microneedling depth, study design, and follow-up duration did not detect a significant interaction but were underpowered and should not be interpreted as confirmatory. Within the short follow-up windows reported (6–16 weeks), both interventions appeared well tolerated, with only mild and transient adverse events; long-term safety, recurrence, and delayed pigmentary complications cannot be characterized from this evidence base. A non-significant pooled difference does not establish therapeutic equivalence in the absence of a pre-specified non-inferiority margin. Microneedling with TXA and microneedling with vitamin C showed comparable short-term outcomes within this limited evidence base. Available data are compatible with a needle depth of ≤1.5 mm being adequate for transdermal delivery. Findings should be interpreted with caution given the modest sample size, predominance of split-face designs, moderate risk of bias in non-randomized studies, and limited power to detect publication bias.

## INTRODUCTION

### Background

Melasma is among the most prevalent acquired hyperpigmentation disorders encountered in dermatology and aesthetic medicine [1-3]. It disproportionately affects women of reproductive age and individuals with darker skin phototypes (Fitzpatrick types III–VI), including populations from Asia, the Middle East, Latin America, and sub-Saharan Africa. Clinically, it manifests as symmetric, irregular macules and patches of light- to dark-brown pigmentation distributed across the centrofacial, malar, or mandibular regions of the face [1]. Although benign, melasma frequently imposes substantial psychological burden—including diminished self-esteem, social anxiety, and depressive symptoms—thereby meaningfully impairing quality of life.

The pathophysiology of melasma is multifactorial, involving a complex interplay of ultraviolet (UV) radiation, hormonal influences (particularly estrogen), genetic predisposition [3], vascular components [4], and low-grade chronic inflammation [1]. These factors converge to induce melanocyte hyperfunction—characterized by increased melanin synthesis and transfer to surrounding keratinocytes—rather than simple melanocyte proliferation [1]. Central to this pathogenic cascade is UV-mediated activation of the plasminogen–plasmin system in keratinocytes, which increases prostaglandin production, thereby stimulating tyrosinase activity [1,2]. Concomitant upregulation of vascular endothelial growth factor (VEGF) contributes to dermal vascular expansion, a recognized feature of treatment-resistant melasma [4].

Given this complex pathogenesis, current management of melasma emphasizes a multimodal approach combining photoprotection, topical depigmenting agents, and procedural interventions [1]. While hydroquinone has historically been the gold-standard topical agent, concerns over long-term adverse effects—including exogenous ochronosis with prolonged use—have renewed interest in alternative agents. Tranexamic acid (TXA) and vitamin C (ascorbic acid) have emerged as clinically prominent alternatives, each operating through distinct but complementary mechanisms of melanogenesis inhibition [5,6].

Tranexamic acid, a synthetic lysine analog, inhibits the plasminogen–plasmin system, thereby suppressing UV-induced keratinocyte-derived paracrine signals that activate melanocytes [1]. It additionally attenuates VEGF-mediated angiogenesis, providing anti-vascular effects relevant to the vascular subtype of melasma [4]. However, its hydrophilic character severely limits passive transdermal penetration, historically necessitating systemic oral administration—which carries thromboembolic risks [7]—or intradermal injection. Vitamin C, conversely, exerts its depigmenting effects primarily through direct competitive inhibition of the copper-binding active site of tyrosinase and interception of dopaquinone in the melanin biosynthesis pathway [5,8], while simultaneously providing potent antioxidant protection and collagen-stimulatory effects [7]. Its hydrophilic nature similarly restricts percutaneous absorption.

Microneedling (MN)—percutaneous collagen induction therapy—has been increasingly adopted as a mechanism to overcome this shared pharmacokinetic limitation [9]. By creating thousands of transient microchannels through the stratum corneum, microneedling dramatically enhances transdermal delivery of both TXA and vitamin C to the viable epidermis and papillary dermis, where melanocyte activity is most relevant to melasma [10]. Beyond its drug-delivery function, microneedling independently activates collagen remodeling, stimulates epidermal turnover, and induces matrix metalloproteinase activity that degrades accumulated melanin deposits [9,10].

### Rationale

The most recent comparative meta-analysis in this area, conducted by Feng *et al*. [11], pooled four studies comparing microneedling with TXA versus microneedling with vitamin C and reported no statistically significant difference in MASI reduction. However, this analysis was critically limited by extreme inter-study heterogeneity (I^2^ = 94%), which fundamentally undermines confidence in its pooled estimate. The authors attributed this limitation to the small number of constituent studies and called for an updated analysis as additional evidence emerged [11]. Since that publication, at least three new comparative studies have been published—including a randomized controlled trial by Vismitha *et al*. [8] and prospective comparative studies by Pazyar *et al*. [9] and Hasan *et al*. [5], which were not captured in the prior synthesis. This expanding evidence base provides a critical opportunity for a more statistically robust and clinically informative analysis [11].

To situate this study within contemporary evidence-synthesis practice, our methodological framework conceptually aligns with recent rigorous comparative evaluations of procedural-plus-topical and device-adjacent treatments across medical disciplines. Similar meta-analytic approaches have been successfully employed to clarify the comparative efficacy of topical therapeutics [12], evaluate outcomes of device-adjacent procedural interventions [13], evaluate comparative outcomes of device-assisted topical treatments in dermatology [14], and compare procedural and pharmacological modalities across specialties [15]. By adopting this robust comparative framework, our analysis seeks to resolve the critical limitations and extreme inter-study heterogeneity (I^2^ = 94%) observed in previous evaluations of microneedling-assisted delivery.

### Objectives

The primary objectives of this systematic review and meta-analysis were: (1) to compare the efficacy of microneedling with TXA versus microneedling with vitamin C in reducing melasma severity, as quantified by standardized changes in the Melasma Area and Severity Index (MASI), Modified Melasma Area and Severity Index (mMASI), or Hemi-Melasma Area and Severity Index (hemi-MASI) scores; and (2) to compare the safety profiles of both interventions, including the incidence and nature of adverse events.

The research question, framed using the Population, Intervention, Comparator, Outcome (PICO) framework, was: *In patients diagnosed with melasma (P), does microneedling (MN) with TXA (I) demonstrate superior efficacy or safety compared with MN with vitamin C (C), as measured by changes in MA*SI scores and the incidence of adverse events (O)?

## Material and Methods

### Registration and reporting

**Figure 1:**
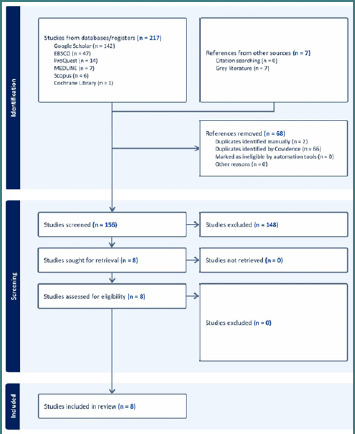
PRISMA flow diagram for the study selection process

This systematic review and meta-analysis were prospectively registered in the PROSPERO International Prospective Register of Systematic Reviews (CRD420251105505) before data extraction. Reporting adheres to the 2020 Preferred Reporting Items for Systematic Reviews and Meta-Analyses (PRISMA) guidelines (Figure 1).

### Information sources and search strategy

Two independent reviewers conducted comprehensive literature searches in MEDLINE (via PubMed), Google Scholar, EBSCO (CINAHL and Academic Search Complete), ProQuest (Health & Medical Collection and ProQuest Central), Web of Science, and ClinicalTrials.gov for publications from January 2015 through December 2025. The search strategy employed Medical Subject Headings (MeSH terms) combined with free-text terms and Boolean operators (AND, OR), structured around the PICO framework.

The search terms included: (‘melasma’ OR ‘chloasma’ OR ‘hyperpigment*’) AND ((‘microneedling’ OR ‘micro-needling’) AND (‘tranexamic acid’ OR ‘TXA’)) AND ((‘microneedling’ OR ‘micro-needling’) AND (‘vitamin C’ OR ‘ascorbic acid’ OR ‘L-ascorbic acid’ OR ‘ascorbate’)) AND (‘efficacy*’ OR ‘effective*’ OR ‘treatment outcome’ OR ‘MASI’ OR ‘Melasma Area and Severity Index’ OR ‘safety*’ OR ‘patient safety’ OR ‘adverse effects’ OR ‘post-inflammatory hyperpigment*’ OR ‘PIH’). Reference lists of included studies and relevant reviews were additionally hand-searched for eligible records.

### Eligibility criteria

#### Inclusion criteria

Studies were eligible if they: (1) employed a prospective comparative design, including randomized controlled trials (RCTs, both parallel and split-face) and non-randomized prospective comparative studies; (2) enrolled adults with a clinical diagnosis of melasma; (3) compared microneedling with TXA (intervention) against microneedling with vitamin C (comparator) in a direct head-to-head fashion; (4) reported quantitative efficacy outcomes using MASI, mMASI, or hemi-MASI; and (5) were published as full-text articles in English.

#### Exclusion criteria

Studies were excluded if they: (1) were duplicate publications; (2) incorporated co-interventions differing between groups; or (3) were rated as having critical risk of bias.

### Study selection and data extraction

Two reviewers independently screened titles and abstracts using Covidence, followed by full-text review against the eligibility criteria. Discrepancies were resolved through discussion or adjudication by a third reviewer. Structured data extraction was performed using a standardized Microsoft Excel form, capturing: study identification (authors, year, country, design), participant demographics (sample size, mean age, Fitzpatrick type), intervention parameters (agent concentrations, needle depth, sessions, follow-up duration and time points), primary efficacy outcomes (MASI/mMASI/hemi-MASI at baseline and endpoint with means ± SDs), adverse event data, and secondary outcomes (patient global assessment, relapse rates).

### Risk of bias assessment

**Figure 2:**
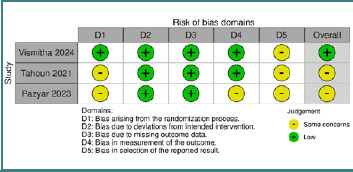
Quality assessment of included randomized controlled trials (RCTs) using the RoB 2 tool

**Figure 3:**
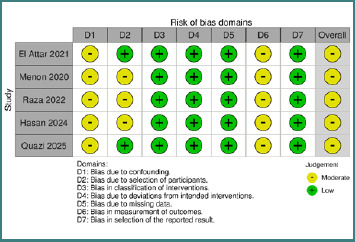
Quality assessment of included non-randomized studies using the ROBINS-I tool

Methodological quality was independently assessed by two reviewers using design-appropriate tools: the Cochrane Risk of Bias Tool Version 2 (RoB 2) for randomized studies and the Risk of Bias in Non-randomized Studies of Interventions (ROBINS-I) for non-randomized prospective studies. Disagreements were resolved by consensus. Results are summarized in traffic-light plots (Figures 2 and 3).

### Statistical analysis

All analyses were performed using Stata (StataCorp, College Station, TX, USA). For the primary continuous outcome, the standardized mean difference expressed as Hedges’ g was calculated to enable pooling across studies using different outcome instruments (MASI, mMASI, hemi-MASI), with negative values indicating a relative benefit of TXA. Effect sizes were computed from within-group change-from-baseline scores (baseline minus endpoint MASI/mMASI/hemi-MASI), not endpoint scores in isolation, so that the standardized mean difference reflects between-arm differences in pigmentation reduction rather than residual endpoint severity. Directionality was harmonized across MASI, mMASI, and hemi-MASI outcomes such that larger positive change scores consistently indicated greater clinical improvement, and the sign of Hedges’ g was set so that a negative value indicates greater improvement in the TXA arm. A random-effects model (DerSimonian and Laird) was the primary analytic approach. Statistical heterogeneity was quantified using the I^2^ statistic and Cochran’s Q test.

The majority of included studies employed split-face designs, in which the two treatment sides of the same participant are inherently correlated. To account for within-person pairing, within-group change scores (baseline minus endpoint) and their associated standard deviations were extracted or imputed for each arm. Where paired correlation coefficients were not reported, an imputed correlation of r = 0.5 was applied as the primary assumption, consistent with established guidance for handling paired data in meta-analysis. A sensitivity analysis was conducted across a range of correlation assumptions (r = 0.25, 0.50, 0.75) to evaluate the robustness of pooled estimates and confidence intervals to this imputation. For studies employing fully independent parallel-group designs, standard unpaired formulae were applied.

Three pre-specified subgroup analyses were conducted and are reported as exploratory rather than confirmatory, given the small number of studies per stratum and wide confidence intervals: (1) microneedling depth (≤1.5 mm vs. >1.5 mm); (2) study design (RCT vs. non-RCT); and (3) assessment time point (baseline, weeks 4, 8, 12). Between-subgroup differences were evaluated using the Qb test. Sensitivity analysis employed the leave-one-out method. Publication bias was assessed with funnel plots, Egger’s regression test, and Begg’s rank correlation test. Given the small number of included studies (*n* = 8), these tests have limited statistical power, and results should be interpreted with caution. For the secondary dichotomous outcome (patient global assessment), pooled risk ratios (RR) with 95% CIs were computed. A two-tailed *P* < 0.05 was considered statistically significant.

## RESULTS

### Study selection

Database searches retrieved 224 records in total (MEDLINE/PubMed: 54; Web of Science: 38; EBSCO: 31; ProQuest: 29; Google Scholar: 68; ClinicalTrials.gov: 4). After automated and manual de-duplication using Rayyan, 156 unique records underwent title and abstract screening; 148 were excluded as irrelevant (wrong population or condition: 61; wrong intervention or comparator: 53; wrong study design: 22; duplicate report: 12). The remaining eight full-text articles were retrieved and assessed for eligibility; all eight met inclusion criteria after full-text assessment, and no full-text articles were excluded at this stage; all eight were incorporated into both qualitative synthesis and quantitative meta-analysis. Hand-searching of the reference lists of all included studies and of relevant prior reviews and meta-analyses on the topic [11,16] was additionally performed; this reference-list search yielded no additional eligible studies beyond those already retrieved through the database searches. The PRISMA flow diagram is depicted in Figure 1.

### Characteristics of included studies

The eight included studies were published between 2020 and 2025 and conducted across five countries: India (*n* = 3), Egypt (*n* = 2), Pakistan (*n* = 1), Iraq (*n* = 1), and Iran (*n* = 1). Six studies employed a prospective split-face comparative design; one was a prospective parallel-group study, and one a randomized single-blind split-face study. The total sample comprised 232 participants (range: 10–40 per study). Where reported, mean participant age ranged from approximately 37 to 40 years; most participants exhibited Fitzpatrick skin types III–V, consistent with the demographic susceptibility to melasma in Asian and Middle Eastern populations. Full study characteristics are presented in Table 1.

**Table 1:** Characteristics of included studies

Study	Country	Study design	*n*	Mean age (yr)	Fitzpatrick type
Quazi *et al*. [17], 2025	India	Prospective split-face comparative	10	NR	IV–V
Hasan *et al*. [5], 2024	Iraq	Prospective parallel-group comparative	40	NR	III–IV
Vismitha *et al*. [8], 2024	India	Randomized single-blind split-face	38	NR	NR
Pazyar *et al*. [9], 2023	Iran	Prospective split-face comparative	34	37.2 ± 8.7	II–IV
Tahoun *et al*. [4], 2022	Egypt	Prospective split-face comparative	30	40.0 ± 6.1	III–V
Raza *et al*. [6], 2022	Pakistan	Non-randomized split-face comparative	30	NR	IV
El Attar *et al*. [7], 2021	Egypt	Prospective randomized split-face	20	39.5 ± 7.0	III–IV
Menon *et al*. [10], 2020	India	Prospective split-face comparative	30	38.2 ± 4.7	IV–V

TXA concentrations ranged from 50 to 100 mg/mL; vitamin C was used at approximately 20% in studies reporting this parameter (Table 2). Microneedling depths ranged from 0.5 to 3 mm, with 1.5 mm most commonly employed. Sessions ranged from 2 to 6 (inter-session interval: 2–4 weeks). Follow-up duration extended from 6 to 16 weeks (Table 3). MASI was the outcome instrument in four studies, mMASI in two, and hemi-MASI in two studies. Adverse events were uniformly mild and transient across all studies, and no serious adverse events were reported (Table 4).

**Table 2:** Intervention characteristics

Study	TXA Concentration	Vitamin C Concentration	MN Depth	Sessions (*n*)
Quazi *et al*. [17], 2025	NR	NR	1.5 mm	3
Hasan *et al*. [5], 2024	NR	NR	1.0 mm	6
Vismitha *et al*. [8], 2024	NR	NR	2–3 mm	3
Pazyar *et al*. [9], 2023	100 mg/mL	100 mg/mL	2–3 mm	3
Tahoun *et al*. [4], 2022	100 mg/mL	20%	1.5 mm	5
Raza *et al*. [6], 2022	100 mg/mL	20%	0.5 mm	3
El Attar *et al*. [7], 2021	100 mg/mL	NR	1.5 mm	6
Menon *et al*. [10], 2020	50 mg/mL	20%	1.5 mm	2

**Table 3:** Outcome measures and follow-up durations

Study	Outcome scale	Follow-up duration	Assessment time points
Quazi *et al*. [17], 2025	mMASI	12 weeks	Baseline, weeks 4, 8
Hasan *et al*. [5], 2024	MASI	12 weeks	Baseline, weeks 2–10
Vismitha *et al*. [8], 2024	MASI	12 weeks	Baseline, weeks 4, 8
Pazyar *et al*. [9], 2023	MASI	12 weeks	Baseline, weeks 2–6
Tahoun *et al*. [4], 2022	hemi-MASI	16 weeks	Baseline, weeks 4, 12
Raza *et al*. [6], 2022	mMASI	6 weeks	Baseline, weeks 2, 4
El Attar *et al*. [7], 2021	hemi-MASI	10 weeks	Baseline, week 10
Menon *et al*. [10], 2020	MASI	8 weeks	Baseline, week 4

**Table 4:** Clinical outcomes and safety profile

Study	Relapse rate	Adverse events reported	Patient global assessment
Quazi *et al*. [17], 2025	NR	Minimal transient side effects	Not reported
Hasan *et al*. [5], 2024	NR	Mild procedure-related pain	Not reported
Vismitha *et al*. [8], 2024	TXA 20%; Vit C 30%	Mild erythema and irritation	Not reported
Pazyar *et al*. [9], 2023	NR	Mild burning during procedure	Reported
Tahoun *et al*. [4], 2022	NR	Mild pruritus, erythema	Not reported
Raza *et al*. [6], 2022	NR	No significant adverse events	Not reported
El Attar *et al*. [7], 2021	NR	Mild post-inflammatory hyperpigmentation	Reported
Menon *et al*. [10], 2020	NR	Mild pruritus, burning sensation	Reported

### Risk of bias assessment

Among the three randomized studies assessed with RoB 2 (Vismitha *et al*. [8], Tahoun *et al*. [4], and Pazyar *et al*. [9]), the overall risk of bias was rated as low to some concerns. Vismitha *et al*. [8] received low ratings across most domains, with some concerns in the selection of reported results. Tahoun *et al*. [4] and Pazyar *et al*. [9] were both rated as having some concerns, particularly in the randomization process and outcome reporting domains. Among the five non-randomized studies evaluated with ROBINS-I (El Attar *et al*. [7], Menon *et al*. [10], Raza *et al*. [6], Hasan *et al*. [5], Quazi *et al*. [17]), the overall risk was predominantly moderate, driven principally by confounding and participant selection domains—inherent limitations of non-randomized designs. No study received a critical risk-of-bias rating. Because five of the eight included studies were non-randomized and predominantly carried moderate risk of bias—primarily from confounding, participant selection, and (in several split-face designs) lack of blinded outcome assessment for MASI, mMASI, and hemi-MASI scoring—the pooled estimate should be interpreted with reduced confidence. Allocation, side-selection, and assessor-blinding bias may operate in the same direction across studies and would not necessarily be reflected in the I^2^ statistic. Outcome assessor blinding status was not consistently reported across the included studies, which is itself a source of measurement bias given the subjective component of MASI/hemi-MASI scoring. Risk-of-bias visual summaries are provided in Figures 2 and 3.

### Primary meta-analysis: melasma severity at treatment endpoint

**Figure 4:**
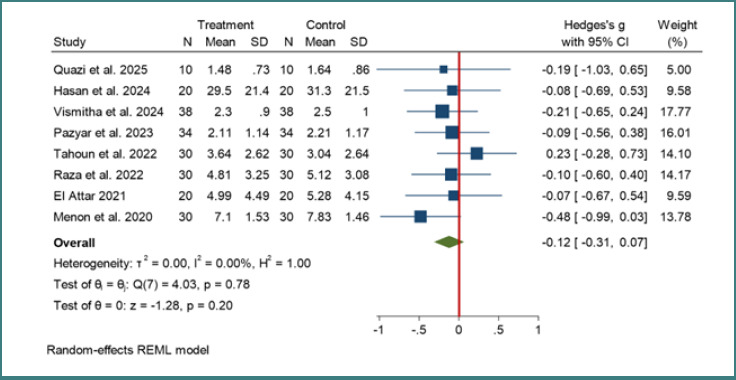
Forest plot of melasma severity reduction at treatment endpoint (overall efficacy)

The pooled analysis of the eight studies under a random-effects model yielded a Hedges’ g of −0.123 (95% CI, −0.311 to 0.065; *P* = 0.201), indicating no statistically significant difference in melasma severity reduction between the TXA and vitamin C groups at the primary endpoint. The direction of the estimate, while non-significant, marginally favors TXA. Critically, there was no inter-study heterogeneity (Cochrane’s Q = 4.03; *P* = 0.776; I^2^ = 0.0%; τ^2^ = 0.0), indicating a high degree of statistical consistency across the included studies. With only eight comparatively small studies, however, low statistical heterogeneity does not by itself imply true clinical homogeneity; the included trials varied in TXA concentration (50–100 mg/mL), vitamin C concentration (variably reported), microneedling depth (0.5–3 mm), session number (2–6), follow-up duration (6–16 weeks), outcome scale (MASI vs. mMASI vs. hemi-MASI), and study design (RCT vs. non-randomized split-face), and these clinical differences should temper interpretation of the I^2^ = 0% finding. The forest plot is presented in Figure 4. Sensitivity analysis (leave-one-out) confirmed the robustness of this finding, with pooled Hedges’ g values ranging from −0.065 to −0.180 upon sequential exclusion of individual studies, none of which materially altered the conclusions.

### Secondary outcome: patient global assessment

A meta-analysis of patient satisfaction data from three studies (Menon *et al*. [10], El Attar *et al*. [7], Pazyar *et al*. [9]), converted to dichotomous responder/non-responder categories, showed a non-significant trend favoring TXA (pooled RR = 1.47; 95% CI, 0.87 to 2.47; *P* = 0.15). This secondary analysis was limited by substantial heterogeneity in satisfaction measurement instruments (I^2^ = 75%; *P* = 0.02).

### Publication bias

**Figure 5:**
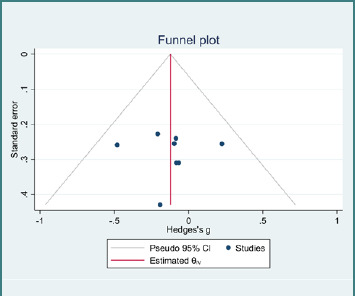
Funnel plot for assessment of publication bias

Funnel plot inspection revealed no apparent asymmetry (Figure 5). Egger’s regression test (*P* = 0.984) and Begg’s rank correlation test (*P* = 1.000) returned non-significant results. However, with only eight included studies, both tests were substantially underpowered to reliably detect asymmetry, and a non-significant result should not be interpreted as confirmation of the absence of publication bias. The possibility of small-study effects or selective non-publication of null findings cannot be excluded.

### Subgroup analyses

#### Microneedling depth

**Figure 6:**
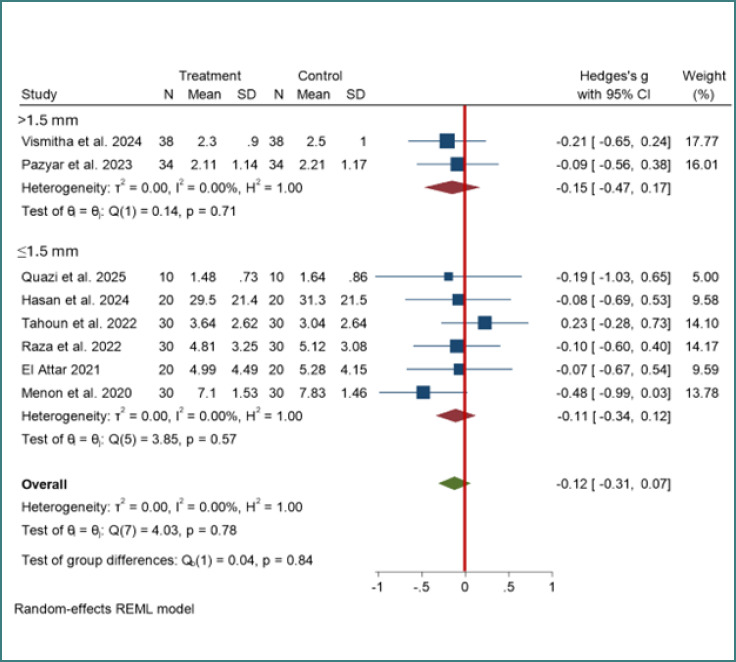
Forest plot of subgroup analysis for microneedling depth

These analyses are exploratory and hypothesis-generating rather than confirmatory. Stratification by needle depth yielded pooled Hedges’ g values of −0.11 (95% CI, −0.34 to 0.12) in the ≤1.5 mm subgroup and −0.15 (95% CI, −0.47 to 0.17) in the >1.5 mm subgroup. Neither subgroup achieved statistical significance, and the between-subgroup test confirmed no significant interaction (Qb(1) = 0.04; *P* = 0.84). The forest plot is shown in Figure 6.

#### Follow-up time point

**Figure 7:**
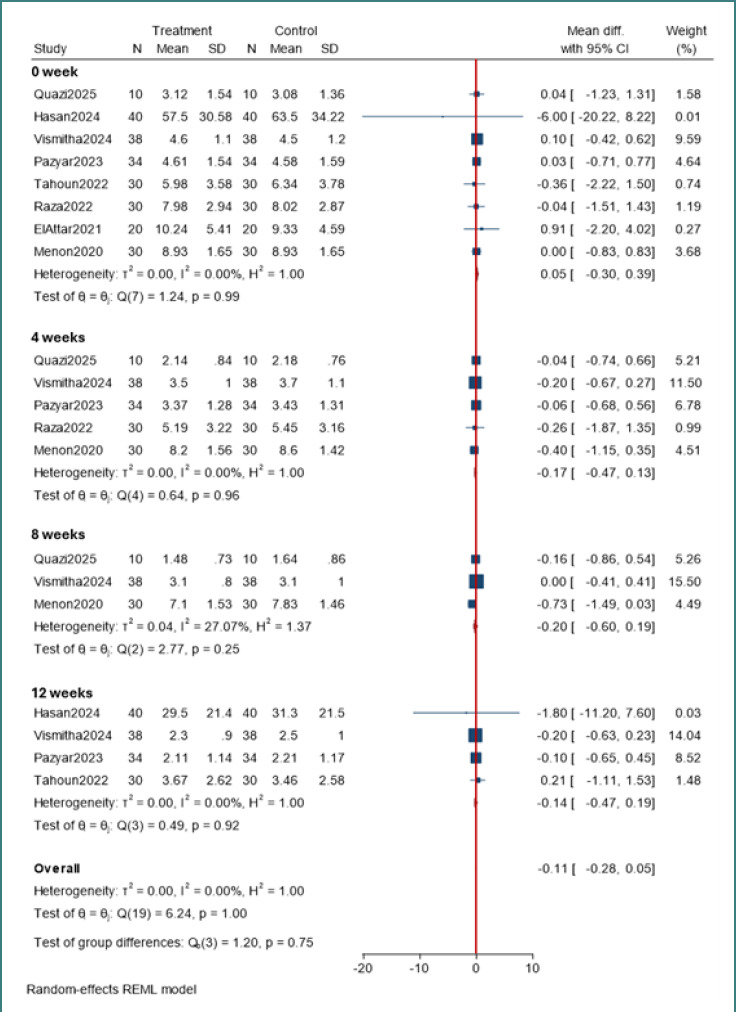
Forest plot of time-course evaluation

At baseline, the pooled mean difference was 0.05 (95% CI, −0.30 to 0.39; I^2^ = 0%), confirming pre-treatment balance. At weeks 4, 8, and 12, pooled mean differences were −0.17, −0.20, and −0.14, respectively, with confidence intervals crossing zero at all time points. The between-subgroup test was non-significant (Qb(3) = 1.20; *P* = 0.75). The forest plot is shown in Figure 7.

#### Study design

**Figure 8:**
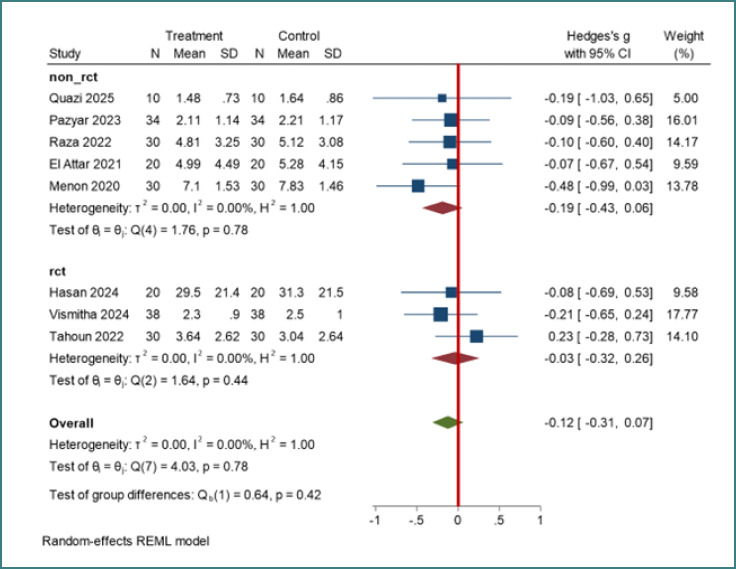
Forest plot of subgroup analysis for RCTs versus non-RCTs

In RCTs (*n* = 3), the pooled Hedges’ g was −0.03 (95% CI, −0.32 to 0.26; I^2^ = 0%), while in non-RCTs (*n* = 5), it was −0.19 (95% CI, −0.43 to 0.06; I^2^ = 0%). The between-subgroup difference was non-significant (Qb(1) = 0.64; *P* = 0.42), confirming consistency across design types. The forest plot is shown in Figure 8.

## DISCUSSION

### Principal findings

This systematic review and meta-analysis provide a comprehensive, up-to-date comparative synthesis of microneedling-assisted topical delivery of tranexamic acid versus vitamin C for melasma, building on a broader evidence base than previously available analyses. The principal finding is that the available evidence did not demonstrate a statistically significant difference between the two interventions in reducing melasma severity at the treatment endpoint, with a pooled Hedges’ g of −0.123 (*P* = 0.201) and zero inter-study heterogeneity (I^2^ = 0%). This finding is robust across all pre-specified sensitivity analyses and subgroup stratifications. The substantially lower heterogeneity compared with the foundational Feng *et al*. [11] meta-analysis (I^2^ = 94%) is likely attributable to the larger evidence base (8 vs. 4 studies), the predominance of split-face designs that control for intra-individual confounders, and the application of Hedges’ g to harmonize heterogeneous outcome instruments. However, it should be noted that if within-person correlations were not fully accounted for in prior analyses, the observed reduction in I^2^ may also partly reflect the statistical properties of paired-data handling rather than exclusively a substantive improvement in study-level homogeneity.

### Mechanistic interpretation

The pharmacological equivalence of TXA and vitamin C when delivered via microneedling is mechanistically coherent when the individual contributions of each agent and the delivery platform are considered together [1,5,6]. Tranexamic acid suppresses melanogenesis through inhibition of the plasminogen–plasmin cascade, reducing prostaglandin-mediated tyrosinase activation [1,2], and additionally targets dermal vascularity through VEGF suppression—an effect particularly relevant in vascular melasma [4]. Vitamin C directly inhibits tyrosinase at the copper-binding site, intercepts dopaquinone to interrupt melanin synthesis [5,8], and provides robust antioxidant protection against UV-induced oxidative stress, with ancillary collagen-stimulatory benefits [7]. The mechanistic relevance of antioxidant activity in pigmentary disorders is supported by experimental evidence demonstrating that ultraviolet (UV) exposure—particularly ultraviolet B (UVB) and ultraviolet A (UVA) radiation—induces the generation of reactive oxygen species, lipid peroxidation, and oxidative cellular injury in keratinocytes and other skin-relevant cell models. Furthermore, antioxidant interventions have been shown to mitigate these UV-induced effects [18,19]. Because UV radiation-induced oxidative stress and downstream pro-inflammatory signaling pathways, including those involving nuclear factor kappa B (NF-κB) and cyclooxygenase-2 (COX-2), are key upstream drivers of melanocyte hyperactivity in melasma, the antioxidant properties of vitamin C are mechanistically aligned with the disease pathogenesis. In contrast, TXA exerts anti-inflammatory and anti-vascular effects, targeting a complementary arm of the same network.

The functional equivalence of these agents in the context of microneedling reflects a key mechanistic principle: both TXA and vitamin C are hydrophilic molecules, and their efficacy depends on transcutaneous delivery that reaches targets at the epidermal–dermal junction [9,10]. Microneedling fulfills this requirement for both agents—it creates microchannels that bypass the stratum corneum barrier, achieving pharmacologically adequate tissue concentrations at the melanocyte compartment [10]. Once at the target site, each agent fully expresses its distinct but convergent inhibitory mechanism, producing clinically comparable outcomes [9,10].

### Clinical implications of subgroup findings

The finding that microneedling depth did not significantly modify comparative efficacy (*P* = 0.84) provides exploratory observations with practical relevance, though the small number of studies per stratum limits their interpretability. The stratum corneum, with an average thickness of 15–20 µm, is the rate-limiting barrier to topical drug delivery. Microneedling to depths of 0.5–1.5 mm reliably penetrates this barrier and creates channels extending into the viable epidermis and superficial papillary dermis—the anatomical compartment where melasma-associated melanocyte hyperactivity resides. Increasing needle depth beyond 1.5 mm accesses deeper dermal layers but does not enhance the bioavailability of either TXA or vitamin C at the melanocyte target, nor does it differentially favor one agent over the other. These observations are consistent with the hypothesis that a needle depth of ≤1.5 mm may be adequate for effective drug delivery in melasma treatment. However, the absence of a statistically significant between-subgroup difference does not establish depth equivalence, and the small number of comparisons across depth strata precludes a definitive procedural recommendation. Prospective studies with pre-specified depth randomization are needed before firm guidance can be issued. In the interim, shallower depths (≤1.5 mm) might be considered on safety grounds given their potential to reduce procedural trauma, post-procedural erythema, and the risk of post-inflammatory hyperpigmentation (PIH) in pigment-prone skin. However, confirmatory evidence from prospective depth-randomization trials is required before any procedural preference can be formally recommended.

The concordance of RCT and non-RCT subgroup results (*P* = 0.42) further reinforces the credibility of the overall pooled estimate. The temporal consistency across the three follow-up time points (weeks 4, 8, 12; *P* = 0.75) demonstrates that neither agent achieves more rapid nor more sustained in-treatment suppression of melanogenesis than the other—a finding that aligns with the known epidermal turnover kinetics of approximately 28–40 days per cycle, during which both agents appear to achieve comparable steady-state inhibition of pigment synthesis.

### Patient satisfaction and individualized decision-making

Although objective efficacy outcomes are statistically equivalent, the non-significant trend toward greater patient-reported satisfaction in the TXA group (pooled RR = 1.47; *P* = 0.15)—consistent with the directional finding of Feng *et al*. [11]—warrants clinical consideration. This trend may reflect the anti-erythematous effect of TXA on vascular components of melasma lesions, a dimension not captured by MASI scoring but perceptible to patients. Additionally, the greater acidity of vitamin C formulations may lead to more procedural burning and irritation, potentially reducing subjective tolerability compared with TXA. While not sufficient to establish clinical superiority, these data suggest that in patients presenting with prominent vascular or erythematous features of melasma, or in those who report intolerance to procedure-related burning, TXA may represent the more appropriate choice. Conversely, in patients for whom skin quality, firmness, and collagen augmentation are co-equal treatment goals, the collagen-stimulatory properties of vitamin C may favor its selection.

### Safety

Within the short-term follow-up windows of the included studies (6–16 weeks), both interventions appeared well tolerated. Adverse events were uniformly mild, transient, and self-limiting—primarily erythema, burning, pruritus, mild procedural pain, and occasional mild PIH—reflecting expected microneedling-associated tissue responses rather than pharmacological toxicity. Adverse events were categorized as mild (grade 1: no intervention required, resolved spontaneously within 72 hours) or moderate (grade 2: required topical management but no procedural discontinuation); no grade 3 or serious adverse events were observed across any of the 232 participants during follow-up periods of 6–16 weeks. For the secondary patient global assessment outcome, a positive response was defined as a rating of ‘improved’ or ‘much improved’ on the investigator- or patient-reported global assessment scale, with ‘unchanged’ or ‘worse’ classified as non-response. The low incidence of PIH, even in studies that predominantly enroll Fitzpatrick type III–V participants, who are phenotypically most susceptible to this complication, underscores the safety advantage of microneedling as a drug-delivery modality compared with ablative energy-based devices. These observations should not be over-interpreted as evidence of long-term safety or durability. Follow-up was limited to 6–16 weeks across all included studies; relapse was reported in only one study, and a pooled sample of 232 participants is underpowered to detect uncommon adverse events such as delayed irritation, persistent erythema, or post-inflammatory hyperpigmentation that may emerge beyond the trial windows. Recurrence of melasma over months to years—clinically the most consequential outcome—cannot be characterized from the present evidence base. Importantly, topically administered TXA via microneedling demonstrated no thromboembolic adverse effects, differentiating it favorably from the systemic risk profile of oral TXA.

Regardless of the procedural modality selected, strict photoprotection is an essential, non-negotiable component of melasma management and must be consistently emphasized during patient counseling. This includes daily application of broad-spectrum sunscreen (SPF ≥50, with UVA and UVB protection), use of sun-protective clothing (including wide-brimmed hats and UV-blocking garments), and active avoidance of peak ultraviolet exposure. UV-driven melanogenesis is the primary driver of melasma persistence and relapse; without consistent photoprotection, the efficacy of any procedural or pharmacological intervention will be substantially attenuated, and recurrence is predictable. Clinicians should reinforce photoprotection at every treatment visit as part of a comprehensive management plan.

### Limitations

Several limitations should be considered in interpreting these findings. First, the absolute sample size (*n* = 232) remains modest, limiting statistical power to detect small but clinically meaningful between-group differences. Individual studies enrolled as few as 10 participants, and pooled confidence intervals—though narrower than previous analyses—still encompass the null value. Second, the geographic homogeneity of the study populations (predominantly South Asian and Middle Eastern with Fitzpatrick types III–V) limits generalizability to populations with lighter (I–II) or darker (VI) skin phototypes. Third, meaningful protocol heterogeneity across studies (TXA concentrations of 50–100 mg/mL; vitamin C concentrations reported variably; needle depths of 0.5–3 mm; sessions of 2–6) reflects real-world variability but may mask clinically important protocol-specific effects. Fourth, follow-up periods of 6–16 weeks are insufficient to assess melasma recurrence—arguably the most clinically important long-term endpoint—and only one study reported relapse rates. Fifth, non-randomized studies carried moderate risk of bias from confounding and selection, and the absence of blinded clinical assessors in most studies introduced potential measurement bias in MASI scoring. Sixth, the pooled estimates rely on an imputed within-person correlation coefficient (r = 0.5) for split-face studies that did not report paired summary statistics. Although sensitivity analyses across r = 0.25–0.75 were conducted, the true correlation is unknown, and the precision of the confidence intervals should be interpreted with this imputation uncertainty in mind. Seventh, with only eight included studies, formal tests for publication bias (Egger’s regression and Begg’s rank correlation) are severely underpowered; the non-significant results of these tests should not be interpreted as ruling out publication bias or small-study effects. Eighth, the Boolean search string was structured to retrieve explicitly head-to-head comparisons and may not have captured eligible studies indexed using alternative terminology or non-comparative study frames; the selection of specific sub-databases within EBSCO and ProQuest may further limit the comprehensiveness of the search.

## CONCLUSION

This systematic review and meta-analysis demonstrate, with high internal consistency and negligible heterogeneity, that microneedling combined with tranexamic acid did not show a statistically significant difference from microneedling combined with vitamin C in reducing melasma severity within this limited evidence base; a non-significant pooled difference is not equivalent to demonstrated equivalence in the absence of a pre-specified non-inferiority margin across multiple subgroup stratifications and follow-up time points. Within 6–16 weeks of follow-up, both interventions appeared well tolerated, and no serious adverse events were reported among the 232 included participants; however, the available studies are underpowered to detect uncommon adverse events, and follow-up was too short to characterize long-term recurrence, delayed irritation, or post-inflammatory hyperpigmentation. The exploratory subgroup data are compatible with a microneedling depth of ≤1.5 mm being adequate for transdermal drug delivery of both agents while minimizing procedural risk, but the small number of studies per stratum precludes any procedural recommendation, and definitive depth guidance awaits prospective comparative trials with pre-specified depth randomization.

Within the limitations of the current evidence base, both agents appear to be reasonable options within a microneedling-assisted treatment paradigm for melasma; the available data do not establish therapeutic interchangeability, and head-to-head non-inferiority trials with pre-specified margins are needed before either agent can be formally designated a first-line standard. Clinical selection between TXA and vitamin C may be guided by individual patient phenotype (with TXA preferred in vascular subtypes), tolerability preferences, co-existing treatment goals (e.g., collagen augmentation favoring vitamin C), and agent availability. Strict photoprotection—comprising daily broad-spectrum sunscreen (SPF ≥50), sun-protective clothing, and avoidance of peak UV exposure—is an essential adjunct to any melasma treatment and should be consistently reinforced in patient counseling, regardless of the procedural modality chosen. Realistic expectations regarding long-term maintenance therapy remain equally important.

Future research should prioritize multi-center, double-blind, adequately powered RCTs with standardized protocols and extended follow-up (≥12 months) to evaluate long-term recurrence rates and maintenance of efficacy. The integration of objective quantitative melanometry and high-resolution dermoscopic imaging as outcome measures would substantially enhance the precision and reproducibility of future comparative analyses.

## Data Availability

Further data is available from the corresponding author upon reasonable request.
